# Vertical distribution and diel vertical migration of krill beneath snow-covered ice and in ice-free waters

**DOI:** 10.1093/plankt/fbt112

**Published:** 2013-11-11

**Authors:** Hege Vestheim, Anders Røstad, Thor A. Klevjer, Ingrid Solberg, Stein Kaartvedt

**Affiliations:** 1 King Abdullah University of Science and Technology, Red Sea Research Center, Thuwal 23955-6900, Saudi Arabia; 2 Department of Biosciences, University of Oslo, Norway, PO Box 1066 Blindern, 0316 Oslo, Norway

**Keywords:** *Meganyctiphanes norvegica*, synchronous and asynchronous DVM, Norway, sea ice, stationary acoustics

## Abstract

A bottom mounted upward looking Simrad EK60 120-kHz echo sounder was used to study scattering layers (SLs) and individuals of the krill *Meganyctiphanes norvegica*. The mooring was situated at 150-m depth in the Oslofjord, connected with an onshore cable for power and transmission of digitized data. Records spanned 5 months from late autumn to spring. A current meter and CTD was associated with the acoustic mooring and a shore-based webcam monitored ice conditions in the fjord. The continuous measurements were supplemented with intermittent krill sampling campaigns and their physical and biological environment. The krill carried out diel vertical migration (DVM) throughout the winter, regardless of the distribution of potential prey. The fjord froze over in mid-winter and the daytime distribution of a mid-water SL of krill immediately became shallower associated with snow fall after freezing, likely related to reduction of light intensities. Still, a fraction of the population always descended all the way to the bottom, so that the krill population by day seemed to inhabit waters with light levels spanning up to six orders of magnitude. Deep-living krill ascended in synchrony with the rest of the population in the afternoon, but individuals consistently reappeared in near-bottom waters already <1 h after the ascent. Thereafter, the krill appeared to undertake asynchronous migrations, with some krill always being present in near-bottom waters even though the entire population appeared to undertake DVM.

## INTRODUCTION

The krill *Meganyctiphanes norvegica* M. Sars, 1857 is a key organism in ecosystems of the northern Atlantic and Mediterranean (Mauchline, 1980; Tarling *et al*., 2010). Its vertical distribution and diel vertical migration (DVM) have been much studied [reviewed by Kaartvedt (Kaartvedt, 2010)]. Generally, adult *M. norvegica* prefer a day time depth between 100 and 500 m (Mauchline and Fisher, 1967) and ascend around dusk to forage near the surface at night. The population normally migrates in synchrony toward the surface in the afternoon. Descent occurs more randomly, though latest at dawn. Most focus has been on DVMs, but vertical distribution related to water clarity (Kaartvedt *et al.*, 1996; Frank and Widder, 2002) and short-term changes related to solar and lunar eclipses (Tarling *et al*., 1999; Strömberg *et al*., 2002) have been reported as well. The conclusion from these studies is that individuals tend to stay shallower as light decreases.

Krill commonly is a main acoustic target at 120 kHz, and many acoustic studies have focused on the distribution and migration behaviour of acoustic scattering layers (SLs) of krill (e.g. Onsrud and Kaartvedt, 1998; Lavoie *et al*., 2000; Everson *et al*., 2007). Most acoustic studies are from hull-mounted transducers on research vessels, although bottom-mounted Acoustic Doppler Current Profilers have been applied in an increasing number of cases (e.g. Tarling, 2003; Brierley *et al*., 2006; Sourisseau *et al*., 2008). Resolution is best close to the transducer (i.e. in upper waters in most cases), while at larger ranges, any deeper living component of the population occurring in lower concentrations becomes hard to separate from the background noise of the echosounder (Klevjer and Kaartvedt, 2006). Therefore, less is known about individuals with distributions deeper than those forming the main SL. Submerged echosounders help to reveal information on the deeper living part of a population, and with their high resolution they even allow study of behaviour at the individual level (Klevjer and Kaartvedt, 2003, 2006, 2011; Onsrud *et al*., 2005).

*Meganyctiphanes norvegica* is an oceanic species, but can also be studied in fjords, making logistics simpler than in open ocean campaigns. In this study, we deployed an upward-looking echosounder on the bottom of the inner part of the Oslofjord, Norway. The echosounder was controlled and powered through a cable to shore, providing unlimited power and storage capacity. This enabled continuous studies throughout the winter, giving information on seasonal patterns in behaviour, as well as responses to unforeseen factors such as freezing of the fjord and subsequent snow on the ice. In addition to information on the main krill SL, the high resolution in deep water offered the opportunity of also addressing the subset of the population that had a deeper distribution than that of the main SL.

## METHOD

The results presented here are from the same campaign as those of Klevjer and Kaartvedt (Klevjer and Kaartvedt, 2011) and Solberg *et al*. (Solberg *et al*., 2012) reporting on the individual swimming behaviour of krill in near bottom waters and the overwintering strategies of the clupeid fish sprat, respectively. These studies also provide information on environmental conditions. Therefore, only a short summary on methods and the environment is summarized here.

### Study site, environmental conditions and sampling

The studies took place from November 2005 to April 2006 at a 150-m deep location (59.792171°N, 10.726776°E) in Bunnefjorden, the inner branch of the Oslofjord, Norway (see Fig. 1 in Klevjer and Kaartvedt, 2011). A mechanical current meter (SD6000) equipped with a temperature probe was deployed together with a conductivity (salinity), temperature and depth (CTD) probe (SD2000) directly above the bottom. The CTD probe gave continuous measurements of temperature and salinity. In addition to the continuous measurements, intermittent sampling campaigns were carried out (in November, December, January and April). Vertical profiles of salinity, temperature and fluorescence were measured using a ship-borne CTD equipped with a fluorometer, and water samples were taken for the measurement of oxygen content. In brief, the water column was characterized by a salinity of 33 and a temperature around 7.5°C in the lower part with slightly fresher and warmer water above (Klevjer and Kaartvedt, 2011). The CTD probe documented the intrusion of new waters on 19 February. Oxygen content in bottom waters was <2 ml O_2_ l^−1^ at the beginning of the winter and >4 ml O_2_ l^−1^ by the end of the study (Klevjer and Kaartvedt, 2011).

**Fig. 1.**
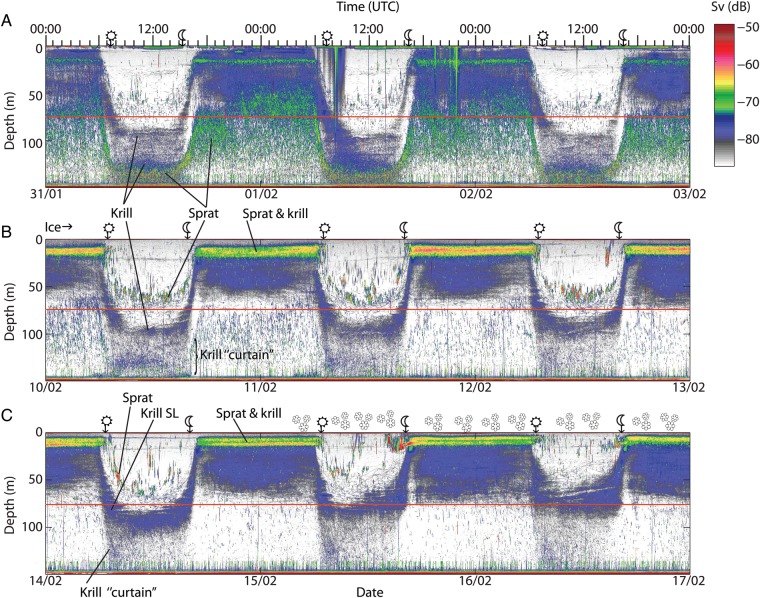

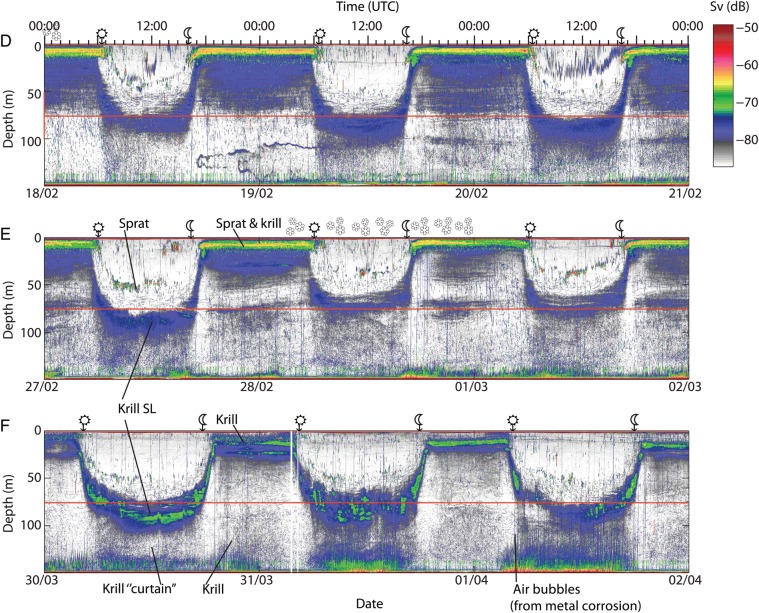
Acoustic records depicting six 3-day periods of representative events occurring during the winter. Time is UTC (h:min) and local time is UMT + 1 (prior to 25 March) and UMT + 2 (after 25 March). 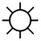 and 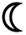 denote time of sunrise and sunset, respectively. Ice was present from 8 February. Heavy snowfall 15–16 and 28 February is denoted with 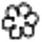. Krill and sprat represent the main acoustic targets, as depicted in the figure. Note the “curtains” of krill beneath the main daytime krill SLs and moreover the shallowing of daytime krill SL following snow cover on the ice. The vertical traces ascending up to 20 m from the bottom are ascribed to bubbles caused by metal corrosion and are not discussed. A red horizontal line is drawn at 75 m to ease readability.

Ice conditions in the fjord were monitored with images obtained with a web camera (1 h resolution), documenting that the fjord was fully ice covered from 8 February to 12 April 2006. Daily measurements of fluorescence at 5-m depth were available from a nearby station in the Oslofjord (Klevjer and Kaartvedt, 2011). Prior to the water renewal on 19 February daily averages of chlorophyll were <0.6 mg m^−3^, and after 19 February it increased to 0.7 mg m^−3^. The spring bloom started around 1 March with progressively increasing chlorophyll values that exceeded 10 mg m^−3^ in mid-March. The April chlorophyll average was 2.8 mg m^−3^ (Klevjer and Kaartvedt, 2011). For this particular study we also collected data on snow cover measured at the Norwegian Meteorological Institute in the nearby city of Oslo. After the fjord became ice covered two major snow falls took place:15–16 and 28 February causing increases in snow depth by 17 and 25 cm, respectively (The Norwegian Meteorological Institute).

During the intermittent sampling campaigns zooplankton were sampled with depth stratified net tows (WP2, 200-µm mesh size equipped with a Nansen release mechanism activated by a drop messenger). Data on the vertical distribution and abundance of copepods in November, December and January are given in Brun (Brun, 2007). In brief, overwintering *Calanus* was present mainly <85 m all 3 months with the highest abundance <112 m (average ± SD between 85 and 112 m and 112, and 145 m was 76 ± 25 and 143 ± 5 ind. m^−3^, respectively). The upper layer (0–20 m) had a high number of *Pseudocalanus* spp. (233 ind. m^−3^) in November, but lower concentrations in December (56 ind. m^−3^) and January (12 ind. m^−3^). *Acartia* occurred in relatively low numbers >20 m in November and December (28 and 27 ind. m^−3^, respectively). *Oithona* spp. was found throughout the water column all 3 months with an overall average of 27 ind. m^−3^. Other copepods occurred only in minor concentrations and the lowest concentration of copepods was found between 20 and 85 m on all dates (Brun, 2007).

Depth stratified pelagic trawling was conducted day and night (43 pelagic hauls in total), targeting acoustic SLs. Data are given in Klevjer and Kaartvedt (Klevjer and Kaartvedt, 2011). In summary, catches were dominated by the krill *Meganyctiphanes norvegica* and the clupeid fish sprat (*Sprattus sprattus* Linnaeus, 1758), which were found in separated SLs. In total 152 l of krill were captured (Klevjer and Kaartvedt, 2011). Two 30-min bottom trawls were performed during daytime 19 December 2005 catching mainly sprat (1079 individuals in total), but also a few fourbeard rockling (*Enchelyopus cimbrus*; 17 individuals), one whiting (*Merlangius merlangus*) and one indet. flatfish besides 0.4-l krill.

### Acoustic studies

An upward-looking, bottom-mounted Simrad EK60 120-kHz echosounder was deployed on the seabed. Ping rates were 1–2 pings s^−1^. The echosounder was cabled to land for power and continuous data transmission to a laptop computer. Data were stored in raw format for later analysis.

Here we present the acoustic data by means of echograms made in MATLAB (MathWorks, Inc.), selecting 3-day periods of representative events occurring during the winter. Also, diel patterns in density/abundance of krill in near-bottom waters were analysed using echo integration in Sonar 5 Pro (Balk and Lindem, 2012) as well as exemplified by acoustic data on individual krill. We furthermore assessed vertical swimming in near-bottom waters using acoustic target tracking (TT) on individual targets (cf. Klevjer and Kaartvedt 2011). In these ways fine-scale information on the deep part of the population was obtained. We integrated acoustic data from February (20–24 February 2006), March (5–10 March 2006) and April (13–18 April 2006). Integration was not performed for the first part of the winter since near-bottom echoes then were dominated by fish (mainly sprat). However, associated with the fjord freezing over sprat ascended to mid-waters the second week of February (Solberg *et al*., 2012), leaving krill as the prevailing near-bottom acoustic target. Integration was made over 5-m depth intervals and 15-min periods and performed over two different thresholds: −65 dB and −85 dB. Acoustic backscattering ascribed to krill was not visible at a threshold of −65 dB at the prevailing concentrations. So to exclude echoes from larger organisms, the results from the −65 dB threshold were subtracted from the −85 dB thresholded data. The density of krill (number of individuals per m^3^) was calculated by dividing the volume backscattering coefficient (linear values of Sv) by the linear value of the average target strength of krill (−72.3 dB) based on *in situ* measurements at 120 kHz in Oslofjorden (Klevjer and Kaartvedt 2006). The results are presented as average number of krill per m^3^ every hour over two different depth intervals (90–110 m and 110–130 m). The 5-day periods from every month (February, March and April) were combined and averaged. As the echosounder was placed on the bottom with the transducer facing upwards ∼0.5 m above the bottom and the transducer in addition has a near-field of ∼1.7 m, this resulted in a blind zone of ∼2.2 m off the bottom.

TT to assess the vertical swimming behaviour was carried out for the same periods as the integration, according to the protocol outlined in Klevjer and Kaartvedt (Klevjer and Kaartvedt, 2011). Since the ability to distinguish between separate targets decreases by range, TT was done for a more restricted depth interval than the integration. Tracks were accepted in the region from 10 to 25 m from the transducer (127.5 to 142.5 m depth), with the majority of tracks being recorded within the first 20 m. Data within each 5-day period were combined in the presentations. Tests for significance of differences in vertical swimming between day and night were made with the Mann–Whitney *U*-test, where tracks after sunset and before sunrise were defined as night, and tracks after sunrise and before sunset were defined as day.

## RESULTS

*Meganyctiphanes norvegica* carried out DVM throughout winter, always linked to the diel light cycle. During daytime in early winter krill was distributed from 90 m and all the way to the bottom. However, fish (sprat) intermingled with the deepest part of the krill distribution and dominated the acoustic records (e.g. Fig. 1A; 31 January–2 February). The fjord was fully ice covered from 9 February, and from ∼10 February, a mid-water SL of krill became established, with highest concentrations at ∼80–100 m, yet with a smaller part of the population spread vertically between this SL and the bottom (Fig. 1B; 10–12 February). Concurrent with snow fall that accumulated on the ice, the upper daytime distribution ascended 20 m (from ∼80 to 60 m) in the course of 15–16 February (Fig. 1C; 14–16 February), subsequently becoming deeper again (Fig. 1D; 18–20 February). No response to the snow fall was evident for krill found in near-bottom waters. The snow did not melt after the first snowfall, but became more packed during a 4-day period with warm daytime temperatures (23–26 February). The krill daytime distribution remained unchanged until the end of February when the distribution became shallower (Fig. 1E; 27 February–1 March) during a period of more snow fall, with the upper part of the SL now at 60 m. As during the previous snow fall, there was no apparent change in deeper waters. Subsequent to 16 March daytime temperatures were above zero every day and the depth of accumulated snow decreased progressively except during a period with wintry showers (sleet) from 27 to 28 March. Krill daytime vertical distribution deepened somewhat throughout March, yet became shallower from 25 to 27 March (no figure). Towards the end of the month the daytime distribution became denser and patchier (Fig. 1F; 30 March–1 April).

The overview echograms show that krill always ascended at sunset (Fig. 1). However, individuals soon dispersed downwards after completing their ascent in the evening. This is seen as increased backscatter in the upper 50–60 m during the first half of February, and for the rest of the study period nocturnal backscatter ascribed to krill appeared dispersed throughout the water column (Fig. 1). This pattern of krill distribution was confirmed by the catches (Fig. 5 in Klevjer and Kaartvedt, 2011), and was also evident in the high-resolution data close to the transducer.

**Fig. 2.**
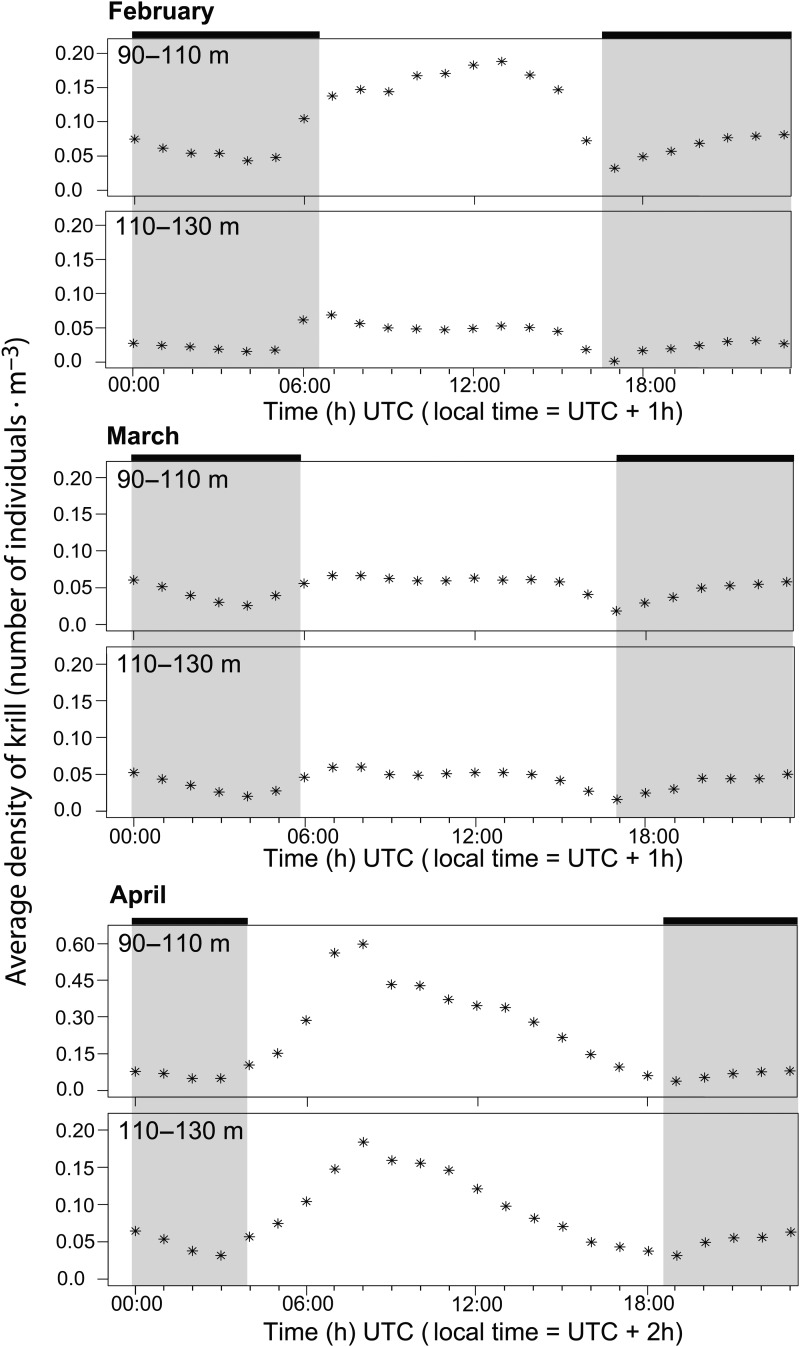
Number of krill per m^3^ and hour at 90–110 m and 110–130 m in February, March and April 2006. A period of 5 days from every month were combined and averaged. The density was calculated using echo integration made over 5-m depth intervals and 15-min periods. Time between sunset and sunrise is shaded grey.

Results from echo integration in the lower part of the water column showed that a number of krill always returned to deep waters relatively soon after sunset (Fig. 2). A subsequent reduction in the abundance of near-bottom krill always occurred after mid-night (Fig. 2), suggesting that there was a second period of nocturnal ascent.

**Fig. 3.**
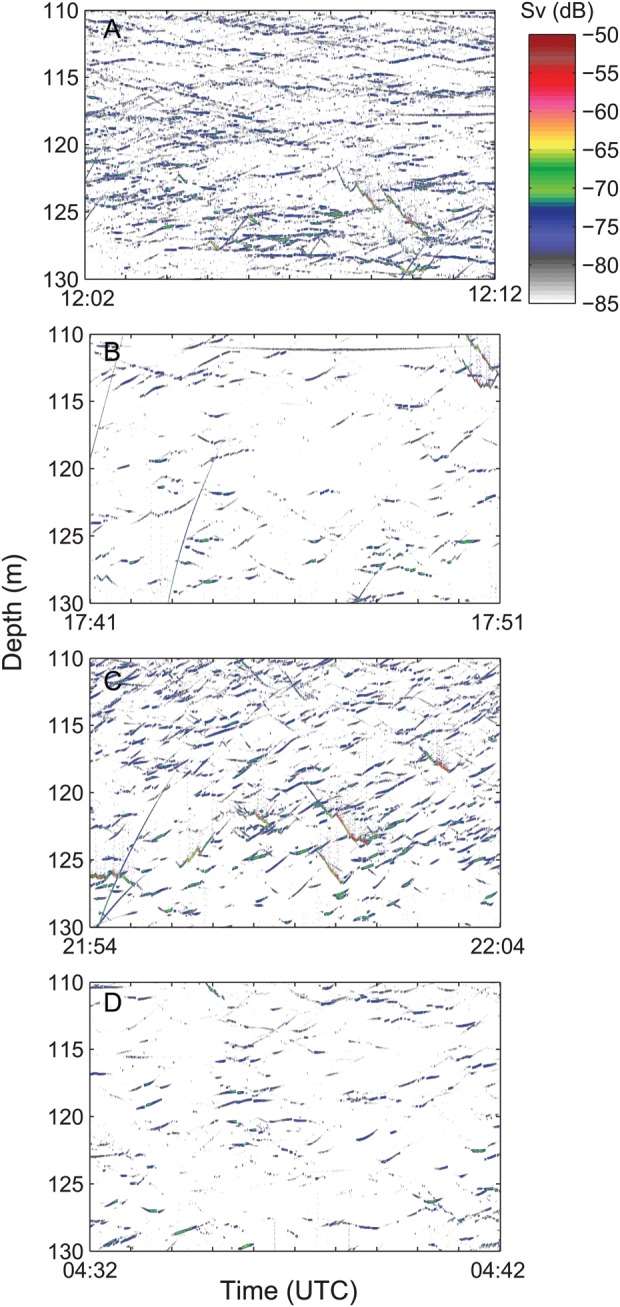
Ten minute echograms from 5 to 6 March, showing individual krill (blue and green lines) in the deeper part of the water column at different times of the day. A few strong (red) targets are sprat. Krill concentrations were higher by day (**A**) and night (**C**) than around dusk (**B**) and dawn (**D**).

The high-resolution data in deep water made it possible to resolve individual krill targets, and even individual krill behaviour, as both upward and downward swimming is evident in the data. In accordance with the findings from the echo integration, the numbers of individuals decreased from day to dusk, subsequently increased at night and decreased again late at night (Fig. 3).

**Fig. 4.**
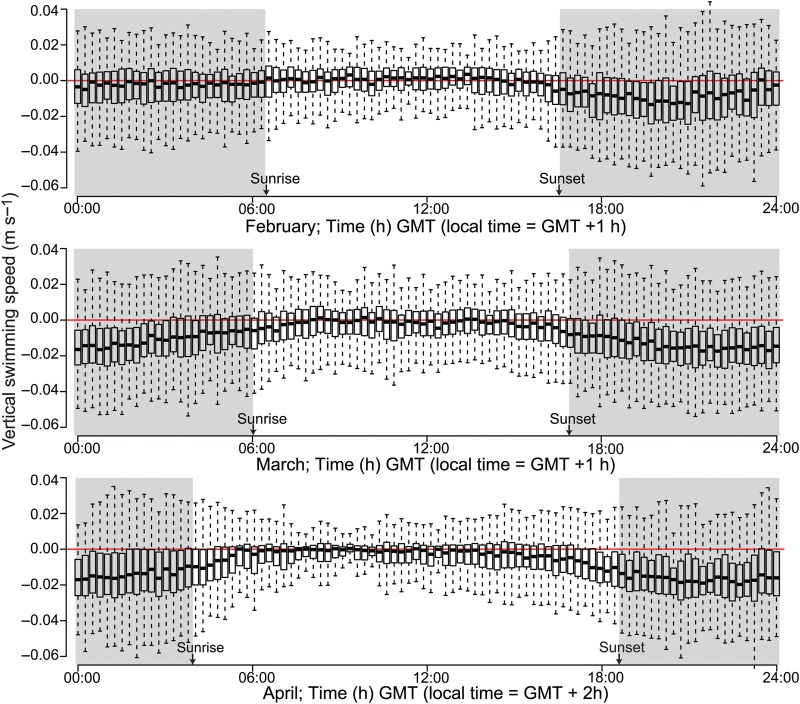
Vertical swimming speed (m s^−1^) of krill in near-bottom waters (10–25 m from the transducer) by time of day for 20–24 February (*n* = 14 713), 5–10 March (*n* = 22 929) and 13–18 April (*n* = 18 285). Each box represents the average for 15 min intervals for 6 (5) subsequent days and spans 50% of the data, the median being depicted by a black line. Whiskers refer to the upper and lower 25%, respectively (excluding outlayers). Positive values refer to upward swimming, negative to downward swimming. Time intervals between sunset and sunrise are shaded.

Data from TT revealed a clear diel pattern in individual vertical swimming behaviour (Fig. 4). Differences in vertical swimming between day and night were highly significant for all periods, both when testing for the 15 min median values and when based on all individual tracks (*P* << 0.001). The median vertical daytime speed was close to zero, with about equal numbers swimming up and down. The vertical swimming increased towards sunset and at night, depicted by a marked increase in records of descending individuals (except for the latter part of the night in February). Normally, <25% of the nocturnal records were of ascending individuals.

## DISCUSSION

*Meganyctiphanes norvegica* carried out DVM throughout the winter. The general pattern comprised synchronous ascent in the evening, subsequent asynchronous nocturnal sinking and rising, before the population appeared to return towards their daytime habitat in the morning. The overall picture of ascent at night showed no evident relation to the abundance of potential food, although we have not addressed the nocturnal distribution in upper waters in detail. Chlorophyll values were low throughout winter, with surface values at the study station varying between 0.2 and 0.4 mg m^−3^ from November to January (Brun, 2007) and with monthly averages from daily measurements at 5-m depth at an adjacent station in the fjord being <0.6 mg m^−3^ until the end of February. The first signs of a spring bloom appeared in early March, peaking in mid-March (Klevjer and Kaartvedt, 2011). Furthermore, zooplankton concentrations, and particularly biomass, rather increased towards the bottom as overwintering *Calanus*, a relatively large copepod and prominent prey of *Meganyctiphanes* in the Oslofjord (Kaartvedt *et al*., 2002) in winter occurred in concentrations of ∼140 m^−3^ in near-bottom waters (140−120 m), but only of ∼10 m^−3^ >85 m (Brun, 2007). Some additional small copepods such as *Acartia* and *Oithona* occurred in upper waters and may have represented potential prey at night [in total ∼100 individuals m^−3^ in December and January (Brun, 2007)]. Net sampling was not done during the period with ice cover, yet concentrations in upper layers likely remained low until *Calanus* started their seasonal ascent, which appears to take place in mid-March in Oslofjorden (Bagøien *et al*., 2001). We did not assess abundance of protozoa that also might have represented krill prey (Schmidt, 2010). Krill may also feed on sediments (Mauchline, 1989; *M. norvegica,* Ligowski, 2000; Clarke and Tyler, 2008; Schmidt *et al*., 2011; *Euphausia superba*) with the possibility of explaining patterns in vertical distribution. Our set-up with a bottom mounted, upward facing echosounder was not appropriate to reveal any such interactions.

Krill daytime vertical distribution changed during the course of winter and we relate this to the light conditions in the fjord, as further outlined below. In addition, the krill SL became denser and patchier at the end of March, possibly related to the onset of the main reproductive season, occurring at this time in northern Atlantic populations of *M. norvegica* (Tarling, 2003).

Freezing of the fjord with subsequent snow cover on the ice led to changes in the krill distribution, presumably mediated through its effect on the light environment. The ice itself seemed to have only a limited influence on the vertical distribution, although the lower edge of the krill-SL seemed to become more distinct subsequent to the first ice cover (Fig. 1). Subsequently, a marked shallowing of the upper edge of the krill SL took place in the course of 15 February, coinciding with a snow fall of 11 cm in Oslo (Norwegian Meteorological Institute). However, the krill distribution deepened again a few days later. The krill may have adapted to the new light conditions under the snow covered ice (cf. Myslinski *et al*., 2005), or a water renewal that occurred on 19 February (Klevjer and Kaartvedt, 2011) might have brought clearer water into the fjord with consequent influence on the under-ice light conditions. Following a second snowfall on 28 February, where snow depth increased by yet another 25 cm, the upper edge of the daytime krill SL shallowed by ∼20 m (Fig. 1E). Unfortunately, we lack measurements of the underwater light field, but a layer of 25-cm snow can reduce transmittance through the ice by one order of magnitude or more (Maykut and Grenfell, 1975; Juhl and Krembs, 2010; Alou-Font *et al*., 2013). With the typical attenuation coefficients found in the Oslofjord, one order of magnitude less incoming light at the surface would correspond to ∼15 m upward displacement of an isolume (authors' unpublished results; http://www.aquamonitor.no/oslofjord; Jerlov, 1968; Onsrud and Kaartvedt, 1998), roughly in accordance with the shallowing of SL recorded here. Previous studies of krill in a nearby arm of the fjord have found light levels at the leading edge of the krill SL to be fairly constant (Onsrud and Kaartvedt, 1998). Relating the shallower vertical distribution subsequent to snowfall to reduction in downwelling light would also be in accordance with previous studies of krill, which have observed corresponding changes in vertical distribution related to more turbid waters (e.g. Kaartvedt *et al*., 1996; Frank and Widder, 2002) or changing incident light (Strömberg *et al*., 2002).

Since our echosounder was located at the bottom, we obtained high-resolution data on the deep part of the population, which often may be lost using hull-mounted echosounders. The daytime echograms (Fig. 1) revealed a “curtain” of weaker backscatter beneath the main SL in mid-waters, representing krill that descended further than the main SL, as shown by the trawling (Klevjer and Kaartvedt, 2011). This backscatter reached all the way to the bottom throughout winter, regardless of the fluctuating upper edge of the SL. The consequent vertical range for the population distribution was at ∼70–90-m depth apparently depending on downwelling light. Therefore, using the attenuation referred to above (one order of magnitude reduction in light per 15 m), the downwelling light experienced by the population of *M. norvegica* in Oslofjorden would have spanned four to six orders of magnitude during daytime. Despite these differences in light experienced the deep-living krill still seemed to ascend the same time in the evening as those higher in the water column. This would be in accordance with Ringelberg (Ringelberg, 1995) suggesting that relative changes in light levels rather than absolute light levels initiate DVM (see Tarling *et al*., 2002).

The high resolution of deep targets also provided evidence for continuous vertical migration behaviour during night. Throughout winter, concentrations in near-bottom waters first decreased at dusk, then increased following “midnight sinking” and subsequently decreased through the latter part of the night (Figs 2 and 3). One interpretation would be repeated (asynchronous) migrations in accordance with the findings of, e.g. Sourisseau *et al*. (Sourisseau *et al*., 2008). Accordingly, the acoustic TT revealed enhanced vertical swimming throughout the night, although primarily as an increase in the number and speed of descending individuals (Fig. 4). This one-way manifestation of enhanced nocturnal vertical swimming activity can likely be explained by reduced detection of ascending individuals. Klevjer and Kaartvedt (Klevjer and Kaartvedt, 2006) did *in situ* TT of *M. norvegica* at 120 kHz and found that krill changing from downward to upward swimming could have its TS reduced by at least 15 dB. Yet, “What goes up, must come down” (Isaac Newton), the latter here detected by TT.

Individuals occupying deeper layers may be subject to epibenthic predators, which might influence distribution and behaviour (Onsrud *et al*., 2004; Hirai and Jones, 2012). However, demersal fish are not abundant in Bunnefjorden, which is frequently hypoxic and even anoxic in the deep waters (Kaartvedt *et al*., 2009). Bottom trawling was only performed in December before ice occurred on the fjord, and the catch of demersal fishes was then limited to a few fourbeard rocklings, one whiting and one flatfish. Also, we did not observe overlap between fish ascending from the bottom and krill in the acoustic records.

In conclusion, krill carried out DVM throughout winter with no evident relation to the distribution of potential food. Migrations were synchronous in the evening and morning and asynchronous at night. The krill adjusted their daytime vertical distribution upwards as ice on the fjord became snow covered, seemingly compensating for ∼one order of magnitude decrease in downwelling light. Still a portion of the population remained in near-bottom water at daytime light levels likely being up to six orders of magnitude lower than for the upper part of the population. These deep-living individuals ascended in synchrony with the rest of the population in the evening. Light evidently plays a central role for the distribution and behaviour of krill, yet there is no single relationship.

## FUNDING

This study was funded by the Research Council of Norway, University of Oslo and King Abdullah University of Science and Technology. Funding to pay the Open Access publication charges for this article was provided by King Abdullah University of Science and Technology.

## References

[FBT112C1] Alou-Font E., Mundy C. J., Roy S. (2013). Snow cover affects ice algal pigment composition in the coastal Arctic Ocean during spring. Mar. Ecol. Prog. Ser..

[FBT112C2] Bagøien E., Kaartvedt S., Aksnes D. L. (2001). Vertical distribution and mortality of overwintering *Calanus*. Limnol. Oceanogr..

[FBT112C3] Balk H., Lindem T. (2012). http://folk.uio.no/hbalk/sonar4_5/index.htm.

[FBT112C4] Brierley A. S., Saunders R. A., Bone D. G. (2006). Use of moored acoustic instruments to measure short-term variability in abundance of Antarctic krill. Limnol. Oceanogr. Methods.

[FBT112C5] Brun H. (2007). Vertical distribution and trophic interactions of krill, sprat and gadoids in the inner Oslofjord during winter.

[FBT112C6] Everson I., Tarling G. A., Bergström B. (2007). Improving acoustic estimates of krill: experience from repeat sampling of northern krill (*Meganyctiphanes norvegica*) in Gullmarsfjord, Sweden. ICES J. Mar. Sci..

[FBT112C7] Clarke A., Tyler P. A. (2008). Adult Antarctic krill feeding at abyssal depths. Curr. Biol..

[FBT112C8] Frank T. M., Widder E. A. (2002). Effects of a decrease in downwelling irradiance on the daytime vertical distribution patterns of zooplankton and micronekton. Mar. Biol..

[FBT112C9] Hirai J., Jones D. O. (2012). The temporal and spatial distribution of krill (*Meganyctiphanes norvegica*) at the deep seabed of the Faroe–Shetland Channel, UK: a potential mechanism for rapid carbon flux to deep sea communities. Mar. Biol. Res..

[FBT112C10] Jerlov N. G. (1968). Optical Oceanography.

[FBT112C11] Juhl A. R., Krembs C. (2010). Effects of snow removal and algal photoacclimation on growth and export of ice algae. Polar Biol..

[FBT112C12] Kaartvedt S., Tarling G. A. (2010). Chapter Nine: Diel vertical migration behaviour of the northern krill (*Meganyctiphanes norvegica* Sars. Advances in Marine Biology.

[FBT112C13] Kaartvedt S., Larsen T., Hjelmseth K. (2002). Is the omnivorous krill *Meganyctiphanes norvegica* primarily a selectively feeding carnivore?. Mar. Ecol. Prog. Ser..

[FBT112C14] Kaartvedt S., Melle W., Knutsen T. (1996). Vertical distribution of fish and krill beneath water of varying optical properties. Mar. Ecol. Prog. Ser..

[FBT112C15] Kaartvedt S., Røstad A., Klevjer T. A. (2009). Sprat *Sprattus sprattus* can exploit low oxygen waters for overwintering. Mar. Ecol. Prog. Ser..

[FBT112C16] Klevjer T. A., Kaartvedt S. (2003). Split-beam target tracking can be used to study the swimming behaviour of deep-living plankton in situ. Aquat. Living Resour..

[FBT112C17] Klevjer T. A., Kaartvedt S. (2006). In situ target strength and behaviour of northern krill (*Meganyctiphanes norvegica*). ICES J. Mar. Sci..

[FBT112C18] Klevjer T. A., Kaartvedt S. (2011). Krill (*Meganyctiphanes norvegica*) swim faster at night. Limnol. Oceanogr..

[FBT112C19] Lavoie D., Simard Y., Saucier F. J. (2000). Aggregation and dispersion of krill at channel heads and shelf edges: the dynamics in the Saguenay - St. Lawrence Marine Park. Can. J. Fish. Aquat. Sci..

[FBT112C20] Ligowski R. (2000). Benthic feeding by krill, *Euphausia superba* Dana, in coastal waters off West Antarctica and in Admiralty Bay, South Shetland Islands. Polar Biol..

[FBT112C21] Mauchline J. (1980). The biology of the euphausiids. Adv. Mar. Biol..

[FBT112C22] Mauchline J., Felgehauer B. E., Watling L., Thistle A. B. (1989). Functional morphology and feeding of euphausiids. Functional Morphology of Feeding and Grooming in Crustacea.

[FBT112C23] Mauchline J., Fisher L. R. (1967). The distribution of the euphausiid crustacean *Meganyctiphanes norvegica*. Ser. Atlas Mar. Environ., Folio.

[FBT112C24] Maykut G. A., Grenfell T. C. (1975). The spectral distribution of light beneath first-year sea ice in the Arctic Ocean. Limnol. Oceanogr..

[FBT112C25] Myslinski T., Frank T., Widder E. (2005). Correlation between photosensitivity and downwelling irradiance in mesopelagic crustaceans. Mar. Biol..

[FBT112C26] Onsrud M. S. R., Kaartvedt S. (1998). Diel vertical migration of the krill *Meganyctiphanes norvegica* in relation to physical environment, food and predators. Mar. Ecol. Prog. Ser..

[FBT112C27] Onsrud M. S. R., Kaartvedt S., Breien M. T. (2005). In situ swimming speed and swimming behaviour of fish feeding on the krill *Meganyctiphanes norvegica*. Can. J. Fish. Aquat. Sci..

[FBT112C28] Onsrud M. S. R., Kaartvedt S., Røstad A. (2004). Vertical distribution and feeding patterns in fish foraging on the krill *Meganyctiphanes norvegica*. ICES J. Mar. Sci..

[FBT112C29] Ringelberg J. (1995). Changes in light intensity and diel vertical migration: a comparison of marine and freshwater environments. J. Mar. Biol. Ass. U.K..

[FBT112C30] Schmidt K., Tarling G. A. (2010). Chapter Five: Food and feeding in northern krill (*Meganyctiphanes norvegica* Sars). Advances in Marine Biology.

[FBT112C31] Schmidt K., Atkinson A., Steigenberger S. (2011). Seabed foraging by Antarctic krill: Implications for stock assessment, bentho-pelagic coupling, and the vertical transfer of iron. Limnol. Oceangr..

[FBT112C32] Solberg I., Klevjer T. A., Kaartvedt S. (2012). Continuous acoustic studies of overwintering sprat *Sprattus sprattus* reveal flexible behavior. Mar. Ecol. Prog. Ser..

[FBT112C33] Sourisseau M., Simard Y., Saucier F. J. (2008). Krill diel vertical migration fine dynamics, nocturnal overturns, and their roles for aggregation in stratified flows. Can. J. Fish. Aquat. Sci..

[FBT112C34] Strömberg J.-O., Spicer J. I., Liljebladh B. (2002). Northern krill, *Meganyctiphanes norvegica*, come up to see the last eclipse of the millennium?. J. Mar. Biol. Ass. UK.

[FBT112C35] Tarling G., Buchholz F., Matthews J. (1999). The effect of lunar eclipse on the vertical migration behaviour of *Meganyctiphanes norvegica* (Crustacea: Euphausiacea) in the Ligurian Sea. J. Plankton Res..

[FBT112C36] Tarling G. A. (2003). Sex-dependent diel vertical migration in northern krill *Meganyctiphanes norvegica* and its consequences for population dynamics. Mar. Ecol. Prog. Ser..

[FBT112C37] Tarling G. A., Ensor N. S., Fregin T., Tarling G. A. (2010). Chapter One: An introduction to the biology of northern krill (*Meganyctiphanes norvegica* Sars). Advances in Marine Biology.

[FBT112C38] Tarling G. A., Jarvis T., Emsley S. M. (2002). Midnight sinking behaviour in *Calanus finmarchicus*: a response to satiation or krill predation?. Mar. Ecol. Prog. Ser..

